# Non-Destructive Inspection Methods for LEDs Using Real-Time Displaying Optical Coherence Tomography

**DOI:** 10.3390/s120810395

**Published:** 2012-07-31

**Authors:** Nam Hyun Cho, Unsang Jung, Suhwan Kim, Jeehyun Kim

**Affiliations:** School of Electrical Engineering and Computer Science, Kyungpook National University, 1370, Sankyuk-dong, Buk-gu, Daegu 702-701, Korea; E-Mails: nhcho@knu.ac.kr (N.H.C.); cester@paran.com (U.J.); shkim1981@gmail.com (S.K.)

**Keywords:** OCT, inspection, LED, real-time, non-destructive

## Abstract

In this study, we report the applicability of two different Optical Coherence Tomography (OCT) technologies for inspecting Light Emitting Diode (LED) structures. Sectional images of a LED were captured using a Spectral Domain OCT (SD-OCT) system and a Swept Source OCT (SS-OCT) system. Their center wavelengths are 850 and 1,310 nm, respectively. We acquired cross-sectional two dimensional (2D) images of a normal LED and extracted sectional profiles to inspect possible wire disconnection that may be present in the LED manufacturing process. The SD-OCT and SS-OCT images were compared with each other in the same sample to study their advantages. The distribution of fluorescence material was observed more clearly with the SD-OCT of 850 nm wavelength, whereas the status of wire connection was clearer in the SS-OCT images with 1,310 nm wavelength. In addition, the volume of the fluorophore space was calculated from the OCT images. This is the first report that a nondestructive optical imaging modality such as OCT can be applied to finding screen defects in LED. We expect this method can improve the inspection efficacy over traditional inspection methods such as Charged Coupled Device (CCD) camera or X-ray instruments.

## 1. Introduction

Optical Coherence Tomography (OCT) has been widely accepted as a non-invasive and high resolution imaging modality for *in vivo* biological specimens. It has been successfully applied for early diagnosis of many diseases originating under superficial areas, including various cancers [1–4]. Although it has limited transmission depth, it can provide high sensitivity in depth resolved images with a high signal-to-noise ratio (SNR). Many commercial OCT products are widely used in ophthalmology as diagnostic equipment [1,2]. Recently, not only medical imaging applications, but also agricultural applications are being actively investigated to search for structural abnormalities that are undetectable with the bare eyes [5–11]. Other interesting applications of OCT are the verification of pearls or counterfeit banknotes [12–14]. OCT is also being spotlighted in industrial fields such as the inspection process for thin film based products. Many attempts to adopt inspection methods using OCT technology in several industrial fields have been reported [15–17]. Machine vision inspection with a Charged Coupled Device (CCD) or visual inspection is widely accepted in Light Emitting Diode (LED) production lines. With these methods, the diagnostic information is limited in two dimensions, and three-dimensional structure information is not promptly feasible. Only in the final production stage, electrical tests are used for inspection of possible defects. Therefore, the manufacturing cost and the missing ratio tends to be increased due to this late inspection stage.

LEDs are lightweight, small in size, semi-permanent and high-speed reactivity. They can be driven with both direct current (DC) and alternating current (AC). Their power can be controlled with the driving current. In addition, they consume less power than traditional lighting. Because of these merits, the application and studies of LEDs have expanded to various fields [18]. Especially after the development of white LED, the demand for commercial products such as the backlight of Liquid Crystal Display (LCD)-TV, car head-lamps, general lighting, and lighting for agricultural products is rapidly increasing. Effective three dimensional inspection methods are required.

In this paper, we examine sectional profiles of LEDs using OCT to solve the problems discussed above. We checked the status of the LED inner structure including wire connection through three dimensional tomographic images. From the acquired OCT images, volume of fluorophors/luminophors is calculated to provide quantitative analysis affecting illuminating intensity and color.

## 2. Experimental Section

### 2.1. LED Operating Principle and Structure

**Figure 1.**
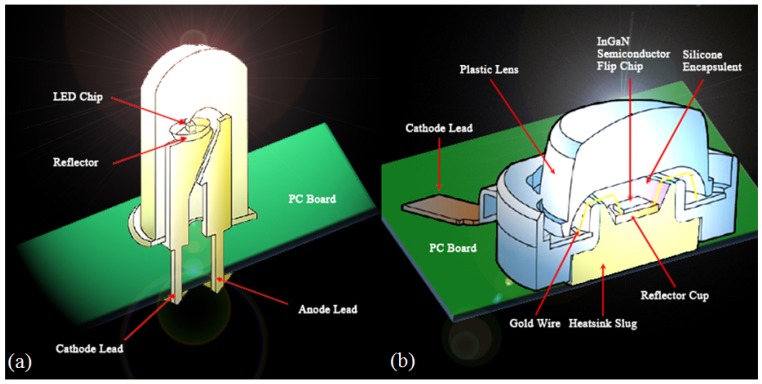
LED structure. (**a**) cylindrical LED; (**b**) chip LED.

A LED is a semiconductor device consisting of a p-n junction diode that emits light when it is biased in a forward direction, called electroluminescence. By the recombination of electrons in the n-type layer and holes at the p-type layer, an LED emits energy corresponding to the energy gap between the conduction band and the valance band. The energy dissipates in forms of a thermal increase or light emission, and the LED corresponds to the latter case [16]. In general, sapphire (Al_2_O_3_) or SiC are used for commercial LED substrates and LED chip are manufactured on those substrates by epitaxial growth. Improving the quantum yield inside the LED active layer is based on control of the growth parameters such as a growth temperature, rate, pressure, and materials used. Crystal defects such as pits which are generated at the early growth phase are diffused to the surface of a completed surface. Those defects are the main reason for decreases in the optical and electrical characteristics of LED devices [19,20]. The wire disconnections during the growth process are hard to find with traditional visual or electrical inspection methods, so if we use the OCT for defect inspection, these defects can be found with this nondestructive method. Figure 1 is an illustration of two LED structures. Figure 1(a) shows the sectional structure of a cylindrical LED. The LED consists of an anode and cathode, which can bias the voltage to the LED; a light-emitting LED chip; a gold bonding wire; fluorophor on the LED chip, which acts as a reflector; synthetic resins filling the LED inside; and a convex lens for the light divergence. This type of LED is less expensive and bigger than the chip type LED, and its driving circuit is easy to make, so it is widely used for general illumination purposes. Figure 1(b) shows the sectional structure of a chip type LED. The chip type is smaller than the cylindrical LED and can be used on a printed circuit board (PCB). It is beginning to make a mark as an alternative LCD backlight, and many other applications are being studied. However, its fabrication and inspection processes are complicated, so reliability of the products is affected by these drawbacks. In this paper, we performed experiments focused on the chip type LED as shown in Figure 1(b).

### 2.2. SD-OCT and SS-OCT Hardware System

**Figure 2.**
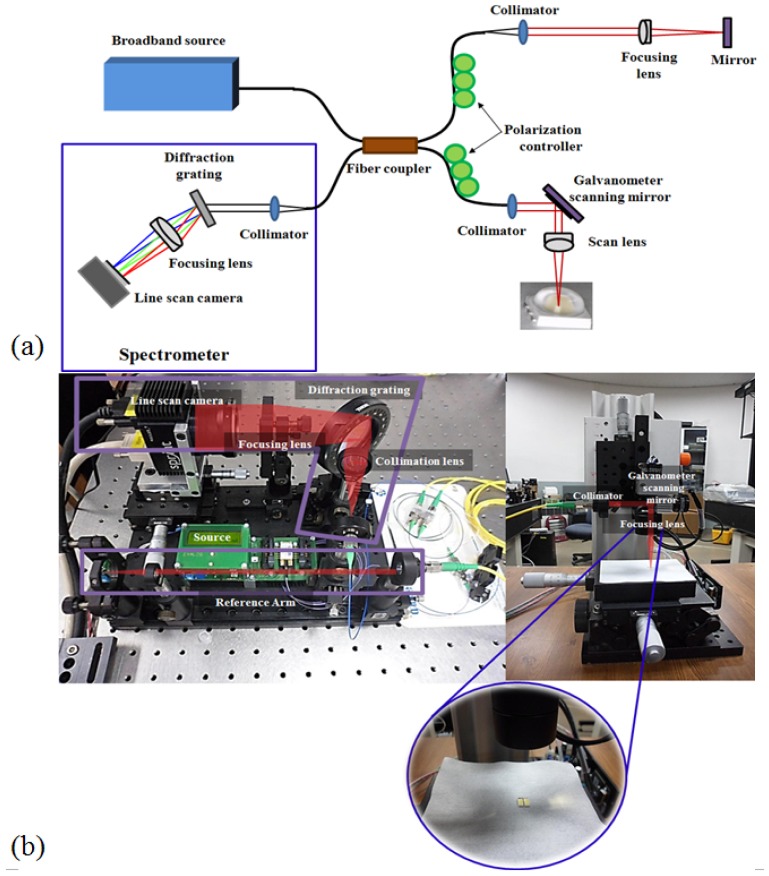
850 nm SD-OCT system. (**a**) Schematic diagram of the SD-OCT system; (**b**) Photograph of 850 nm SD-OCT system and sample arm optic setup.

The schematic diagram of the developed SD-OCT system is shown in Figure 2(a). A 12-bit Complementary Metal-Oxide Semiconductor (CMOS) line-scan camera (Sprint spL2048-140k, Basler AG, Ahrensburg, Germany) with a 70,000 line/s effective line rate in the 2048 pixel mode was used as a detector in the SD-OCT system. With the junction of a EXS8510-2411 Super Luminescence Diode (SLED; λo = 850 nm, Δλ = 55 nm, Exalos Ltd., Schlieren, Switzerland) as a light source, a fiber-based interferometer was implemented. The light source was split into sample and reference arms with the latter terminated by a stationary mirror. A probe at the end of the sample arm delivered light to, and collected back-scattered light from, different depths in the sample. B-mode scanning was performed using a galvanometer scanning mirror (GVS002, Thorlabs, Newton, NJ, USA) at the back focal plane of the objective lens on the sample arm. The output of the line camera was connected to a personnel computer (PC) through a PCIe-1433 frame grabber (NI, Austin, TX, USA) which has a maximum 850 MB/s bandwidth over two camera link cables. The galvanometer scanning mirror was driven by the PC with a PCIe-6321data acquisition board (DAQ, NI) which can provide two analog outputs.

Figure 2(b) is a photograph of the SD-OCT system, consisting of the spectrometer, the reference path, the source, and the polarization controller. The right side picture in Figure 2(b) shows the customized sample path for easy access from the LED. To customize the sample path, we used a micro-stage that can be controlled minutely in three dimensional directions.

**Figure 3.**
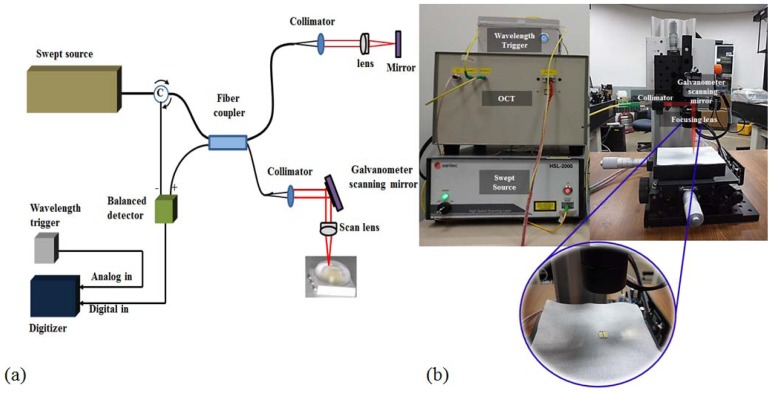
1,310 nm SS-OCT system. (**a**) Schematic diagram of the SS-OCT system; (**b**) Photograph of 1,310 nm SS-OCT system and sample arm optic setup.

The schematic diagram of the developed SS-OCT system is shown in Figure 3(a). The center wavelength (λ_C_) of the light source (Santec HSL-2000, Komaki, Japan) is 1,310 nm, while the Full Width Half Maximum (FWHM) is 110 nm, and the maximum line scan speed is 20 kHz. We used a balanced detector (Thorlabs PDB110C) as a light detection tool whose effective bandwidth is from 800 to 1,650 nm, and the electronic bandwidth is 100 MHz. A PCT-5122 digitizer (NI) which has two analog input channels, one trigger channel at a maximum sampling rate of 100 MHz, was used for signal processing. A PCI-6221 DAQ board (NI) with a maximum sampling rate of 833,000 samples per second was used for driving the galvanometer scanning mirror (Thorlabs GVS002). The axial and lateral resolution is 4 μm and 58 μm, respectively. The lateral scanning range is flexibly set to cover the sample area.

Figure 3(b) displays the exterior of the SS-OCT system that contained the optical setup for the light source, balanced detector, and the reference path as mentioned above. All other controllers and power supplies were included in the system. The right side picture in Figure 3(b) displays the same sample arm setup to scan longitudinal and transverse directions with the SD-OCT system.

### 2.3. OCT Software System and Performance

**Figure 4.**
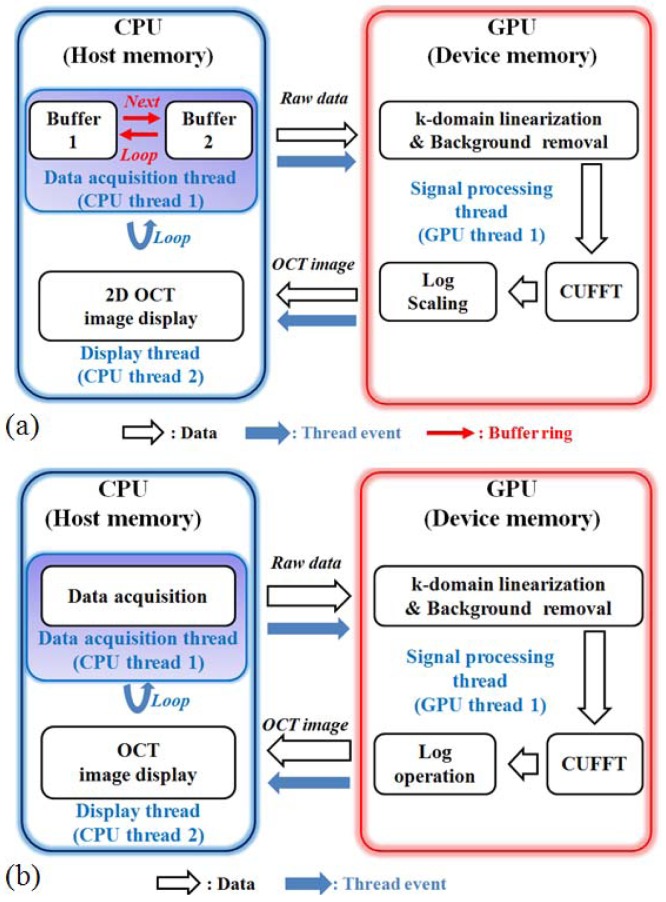
Program block diagram. (**a**) SD-OCT Program block diagram; (**b**) SS-OCT Program block diagram.

The driving software for both the SD and SS-OCT systems were programed using Compute Unified Device Architecture (CUDA) version 3.2 for Visual Studio 2008 and Graphics Processing Unit (GPU) programming. Figure 4(a) is the block diagram of the SD-OCT program. To improve the data acquisition speed, we used a personal computer with two main memories (RAM) as buffers. Figure 4(b) is the structure diagram that shows the procedure to display images from the memory assigned to the digitizer. During this procedure, the data are acquired from the digitizer and copied to the RAM. We adapted the GPU to provide fast image processing and displaying, which are important issues in the fault inspection environment. With GPU processing, we could realize the real-time display feature after performing massive data processing, including k-domain linearization, background removal, Fast Fourier Transform (FFT), and log scaling processes [21–23].

**Figure 5.**
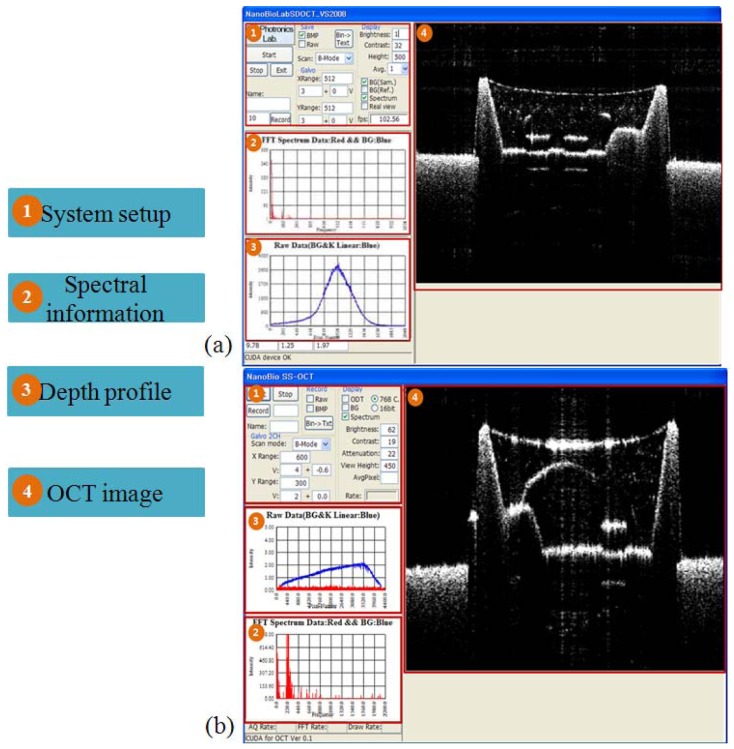
Program UI. (**a**) 840 nm SD-OCT program UI; (**b**) 1,310 nm SS-OCT program UI.

Figure 5 shows the user interface (UI) of the SD and the SS-OCT systems. Figure 5(a) is the UI of SD-OCT and Figure 5(b) is that of SS-OCT. The number “(1)” in the picture represents the system setup part consisting of control panels displaying the current program speed, image saving, background removal, adjusting the brightness and the chroma, driving the galvanometer scanning mirror, and scan mode change. At the spectral information part ((2)), the detailed information after FFT and changing to log scale can be seen. The spectrum information before FFT is shown at the depth profile part ((3)). The OCT image part ((4)) shows the real-time display of a 2D image based on the acquired data. The system speed is 120 frame/s in the SD-OCT system with the image size of 1,024 × 512 pixel, and 20 frame/s in the SS-OCT system (2,500 × 200 image size). The reason for the slower frame rate in the SS-OCT system is due to limitation of the data transfer speed from the digitizer.

## 3. Results

**Figure 6.**
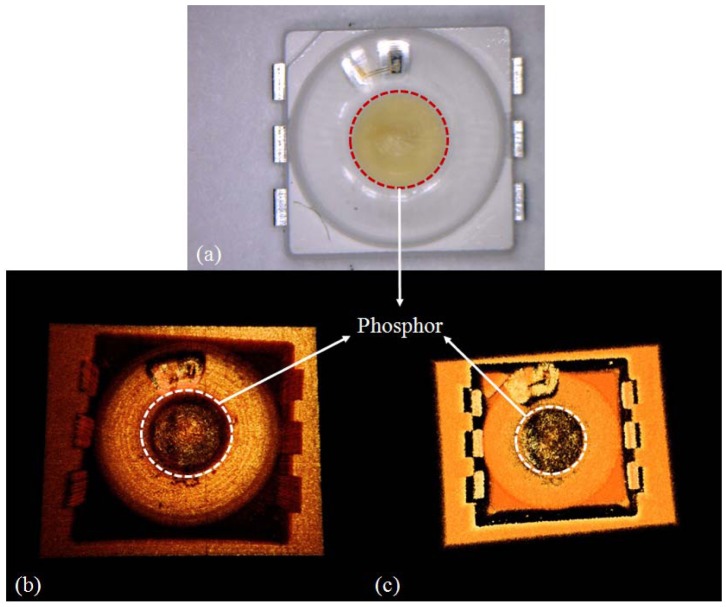
Circle chip LED. (**a**) Microscope top view image; (**b**) 850 nm SD-OCT top view 3D image; (**c**) 1,310 nm SS-OCT top view 3D image.

Figure 6 shows the image of the circle chip LED. Figure 6(a) is the 20X magnified image of the circle chip LED using a digital microscope (Dino-lite AM3013, New Taipei City, Taiwan). Figure 6(b,c) are OCT images taken with SD-OCT (850 nm center-wavelength) and with SS-OCT (1,310 nm center-wavelength), respectively. We reconstructed the 3D image from 512 2D images. The 3D image displays the entire internal structure of the LED including the morphological shape of the fluorophore. Also, the status of wire connection appeared in the upper part of the image. The fluorophore is more clearly observable in the SD-OCT image compared to the SS-OCT as shown in Figure 6(b,c).

**Figure 7.**
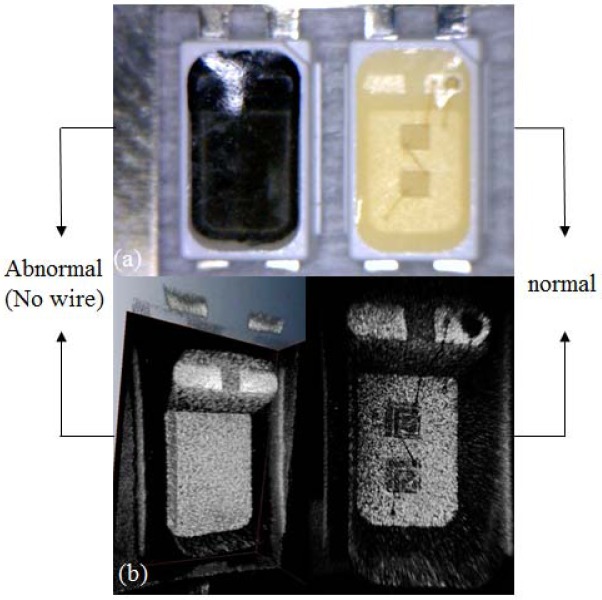
Comparison of digital microscope and OCT images of faulty and normal LED. (**a**) Abnormal (no wire) and normal LEDs digital microscope image; (**b**) Abnormal (no wire) and normal OCT image.

We compared an abnormal LED with a normal one using digital microscope and OCT as shown in Figure 7. The images, shown in Figure 7(a), represent the digital microscope views of the abnormal and normal samples. The left black LED is the abnormal sample without wire connections. The inner structures are also observed with OCT as shown in the Figure 7(b). We could confirm the wire-connection status of abnormal and normal LEDs with OCT imaging. Through this experiment, OCT imaging can detect the status of LEDs by observing the wire-connection status.

**Figure 8.**
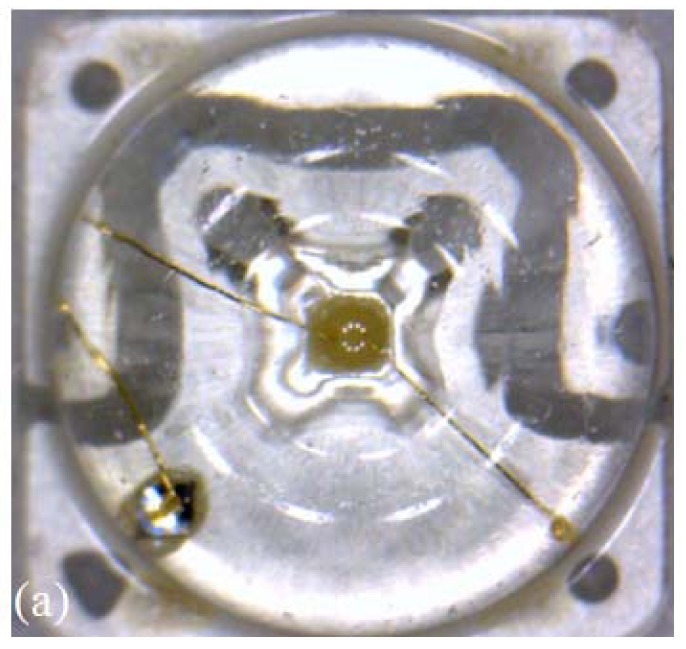

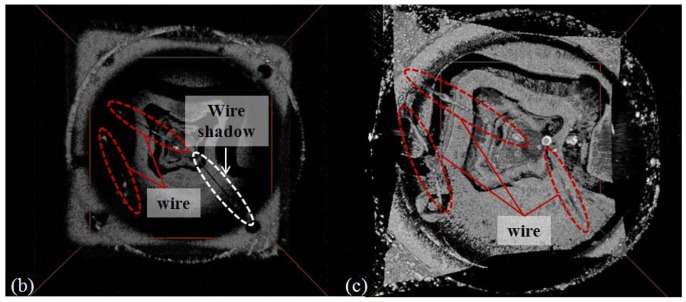
Circle chip LED. (**a**) Microscope top view 3D image; (**b**) 850 nm SD-OCT top view 3D movie 1; (**c**) 1,310 nm SS-OCT top view 3D movie 2.

Figure 8 is the experimental result with a transparent chip LED for confirming the status of wire connection. Figure 8(a) is the microscope image of a transparent chip LED with the same equipment and magnification as previously used. Figure 8(b) is the 3D reconstructed animation with SD-OCT (850 nm center-wavelength). With this system, the electric wire presence is observable, but not enough to decide if there is any discontinuity. However, the SS-OCT approach provides better SNR for the wire inspection as shown in the 3D reconstructed animation Figure 8(c). On the contrary, the image of fluorophore beneath the wire is dimmer than the images taken from SD-OCT.

**Figure 9.**
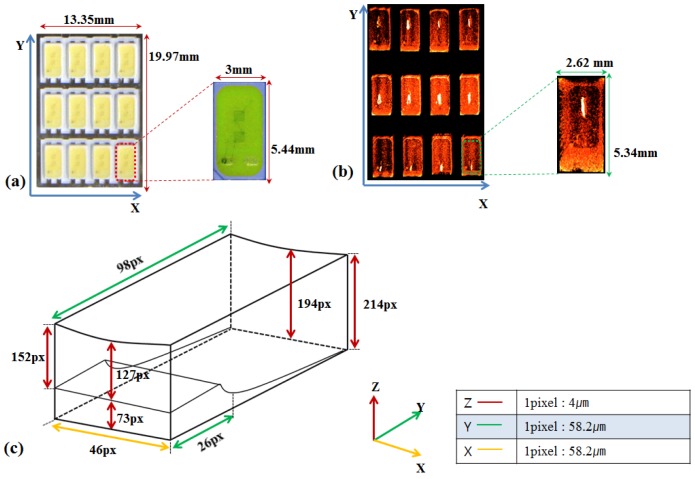
Microscope and OCT images of LED array (**a**) The microscope image of LEDs; (**b**) The OCT image of LEDs; (**c**) the inner frame view of LED.

To examine the amount of fluorophore covering the chip, OCT scans of 12 LEDs are performed and compared with the microscopic image. Importantly, the volume of the fluorophore area is calculated because it is crucial to constant illumination and should be carefully monitored in manufacturing process. Figure 9(a) is the microscopic view of the sample containing 12 LEDs with the width of 13.35 mm and the height of 19.97 mm. Each LED size is 3 mm in width and 5.44 mm in height, respectively. Figure 9(b) is the reconstructed 3D OCT image of the entire sample that is taken in a burst scan without any rearrangement during the scan. The space of the fluorophore part was extracted from the single entire OCT image and the volume was calculated. Figure 9(c) graphically shows the inner part of a LED indicating the fluorophore space. This inner part is also extracted from the obtained OCT image. The unit in Figure 9(c) is in pixel and each pixel size in x, y, and z direction is 4 μm, 58.2 μm, and 58.2 μm, respectively.

Table 1 is the result of volume calculation based on OCT imaging shown in Figure 9. The average volume of the fluorophore was about 52.02 mm^3^. The non-destructive and quantitative measurement of the fluorophore volume has not been practical, but this result indicates that OCT may take an important role in the 3 dimensional volume inspection in the LED manufacturing process.

## 4. Conclusions

In this paper, we nondestructively observed the sectional profile of chip type LEDs, which are commonly used in a variety of fields, with real-time imaging OCT to increase screening efficacy in manufacturing lines. We compared the imaging quality of an SD-OCT with 850 nm center-wavelength to that of the SS-OCT with 1,310 nm center-wavelength to determine their efficiency as screening tools. Various sectional images of normal chip LEDs were obtained. From those 2D and 3D images, we focused on verification of wire disconnection and the status of the fluorophore. The images taken from each OCT system were compared with each other. The distribution of fluorophore was observed well in the SD-OCT image with an 850 nm center-wavelength, whereas the status of the wire connection was confirmed well in the SS-OCT image with a 1,310 nm center-wavelength. Through the comparison of the OCT images to the digital microscope image, we confirmed the reliability of OCT screening. The volume of the fluorophore in each LED is also quantitatively measured using OCT. This measurement would be important enhancement of the three dimensional inspection in the LED manufacturing process. This measurement has not been easily feasible using conventional techniques due to either 2D limitations or poor depth resolution/range. As far as we are aware, this is the first report that a nondestructive optical imaging modality, such as the OCT, can be applied to screen for defects in LEDs. We expect this method may contribute improvement of the inspection efficacy over any traditional inspection methods such as CCD camera or the X-ray instruments.

## Figures and Tables

**Table 1.** The average volume of the fluorophore space in the LED (N = 12).

**N**	**X (mm)**	**Y (mm)**	**Z (mm)**	**Volume (mm^3^)**
1	2.851	4.831	3.960	54.537
2	2.619	5.587	3.680	53.848
3	2.793	5.703	3.600	57.348
4	2.560	4.714	3.840	46.342
5	2.619	5.412	3.640	51.599
6	2.560	5.529	3.560	50.389
7	2.560	4.597	3.920	46.139
8	2.502	5.703	3.600	51.373
9	2.677	5.878	3.520	55.390
10	2.619	4.830	3.760	47.569
11	2.560	5.761	3.680	54.280
12	2.619	5.587	3.680	53.848

**Average**	2.628	5.344	3.703	52.021
